# Smart phone addiction and its mental health risks among university students in Jordan: a cross-sectional study

**DOI:** 10.1186/s12888-023-05322-6

**Published:** 2023-11-07

**Authors:** Sawsan M A Abuhamdah, Abdallah Y Naser

**Affiliations:** 1 Department of Biopharmaceutics and Clinical Pharmacy, School of Pharmacy, The University of Jordan, P.O.Box: 13380, Amman, 11942 Jordan; 2 Department of Pharmaceutical Sciences, College of Pharmacy, Al Ain University, P.O.Box: 112612, Abu Dhabi, UAE; 3 Department of Applied Pharmaceutical Sciences and Clinical Pharmacy, Faculty of Pharmacy, Isra University, Amman, Jordan

**Keywords:** Addiction, Mental, Mobile, Smart phone, Students

## Abstract

**Background:**

Addiction to smart phones is classified clinically as behavioral addiction resulted from an excessive problematic usage of smart phones that effect the daily life of the users. Therefore, this study aims to explore the prevalence of smart phone addiction, its associated psychological distress risk, and its associated predictors among university students in Jordan.

**Methods:**

Between November 2022 and January 2023, a cross-sectional online survey study was conducted in Jordan. In this study, we used previously developed questionnaire instruments, the psychological Distress scale of Kessler and the Smartphone Addiction Scale. A score of 30 was used to identify the dummy variable in the binary logistic regression analysis to identify predictors of severe psychological distress, and smartphone addiction score of 38.7 was used to to identify predictors of smartphone addiction.

**Results:**

A total of 2337 university students participated in this study. The mean psychological distress score for the study participants was 30.0 (SD: 8.9). More than half of the study participants (59.1%) had a psychological distress score of 30 and above, which indicates a severe mental disorder state. More than half of the study participants (56.7%) had a smartphone addiction score of 30 and above, which reflects a smartphone addiction state. Females, divorced, those who feel that their mental abilities have been negatively affected by the use of smart phones, those who feel that using smartphones has affected their sleep and made it harder to fall asleep, and those feel that everything requires effort and fatigue, and they do not want to do any activity that requires effort were more likely to have severe psychological distress compared to others (p < 0.05). Females, those who feel that using smartphones has affected their sleep and made it harder to fall asleep, and those feel that everything requires effort and fatigue, and they do not want to do any activity that requires effort were more likely to be smartphone addicted compared to others (p < 0.05).

**Conclusion:**

Mental diseases are a major public health concern in Jordan, especially among university students. Females, those who thought smartphone usage hurt their mental capacities, and those who had trouble sleeping and fatigue were more likely to develop serious psychological discomfort and smartphone addiction. Smartphones are indispensable, but excessive use can lead to addiction and harm university students’ mental health.

## Background

Smart phones are defined as a personal device that indicate social identity and status for the user and its main difference of any other mobile phone is that it ensures a continuous connection to the internet and provide several services including sociability, entertainment, information finding, time management, coping strategies, and social identity maintenance [1]. Indeed, smart phones increased development during the past few years had led to facilitate the access to smart phones and increased the usage of smart phones among a wide range of population throughout the world [2]. Meanwhile, using the term addiction for an increase usage of smart phones is highly debatable [3]. However, excessive usage of smart phones leads the addiction of usage to be a threatening worldwide issue [4].

The term “smartphone addiction” refers to a phenomenon in which individuals display excessive, compulsive, or harmful behaviors associated with their utilization of smartphones [5–7]. This addiction is typified by an individual’s lack of ability to regulate their smartphone utilization, even in instances when it yields adverse repercussions on their day-to-day existence, interpersonal connections, professional endeavors, or general state of welfare [5–7]. Addiction to smart phones is classified clinically as behavioral addiction resulted from an excessive problematic usage of smart phones that effect the daily life of the users leading to (inoccupation, compulsive behavior, control deficiency, functional deterioration, deprivation, and tolerance) [5, 7], However, there is no specific criteria to diagnose or to determine the addiction of smart phone [7].

The rate of smart phone addiction was in a significant increase recently especially among university students, where the rate of smart phone addiction among university students in turkey accounted for 34.6% [8], and 97.8% in Iran [9], and 44.0% in south India [10], where the results revealed a significant wide range between the studies, and this difference might be due to the usage of different smart phone addiction scales [11], and the usage of smart phone applications as base for the addiction scale [12].

The extreme inappropriate usage of smart phones results in multiple health and physical issues including sleeping disorders for either falling asleep or maintain the sleep [4], eye health [13, 14], musculoskeletal system [15], as well as it results in traffic and other sever accidents [16]. Meanwhile, smart phone addiction can also cause mental, behavioral, and social issues, and it is related negatively with multiple concepts of health and well-being [17], where smart phone addiction causes attention deficit and maladaptive behavior issues, interferes with school and employment, lowers performance in school, and decreases in-person social contacts [18]. Therefore, this study aims to explore the prevalence of smart phone addiction, its associated psychological distress risk, and its associated predictors among university students in Jordan.

## Methods

### Study design

Between November 2022 and January 2023, a cross-sectional online survey study was conducted in Jordan.

### Sampling strategy

Eligible individuals were identified and invited to participate in the study using convenience sampling. Facebook and WhatsApp were used to reach out to university students and invite them to participate in this study. The questionnaire link was distributed among different social media pages and WhatsApp groups that are of interest to university students. Consequentially, written consent was not required, as all subjects gave their informed consent voluntarily. Detailed descriptions of the study’s goals and objectives were provided at the outset of the questionnaire. The inclusion criteria were university students who resided in Jordan, were at least 18 years old, and were enrolled in any level of study. Participants under 18 or unable to read or comprehend Arabic were not invited to participate. The required sample size from each study population was 385 participants, based on a confidence interval of 95%, a standard deviation of 0.5, and a margin of error of 5%.

### Study tools

In this study, we used the Arabic version of previously developed questionnaire instruments to investigate smart phone addiction and its associated psychological distress risks among university students in Jordan. In this investigation, the psychological Distress scale of Kessler and the Smartphone Addiction Scale were utilized [11, 19]. The first part of the questionnaire examined patients’ demographics (age, gender, marital status, study level, faculty, and monthly income). In addition, the first part asked the participants whether they feel that their mental abilities have been negatively affected by the use of smart phones, whether they feel that using smartphones has affected their sleep and made it harder to fall asleep, and whether they feel that everything requires effort and fatigue, and they do not want to do any activity that requires effort. The second part examined the prevalence of psychological distress among the study participants using Kessler psychological Distress scale. The Kessler psychological Distress scale is a 10-item questionnaire designed to produce a global measure of distress based on queries about anxiety and depressive symptoms experienced within the past four weeks. It is a valid and reliable measurement of psychological distress and is available in Arabic [20]. It quantifies psychological distress. The patient identification identifiers The cumulative score on the Kessler Psychological Distress Scale (K10) is determined by adding 10 responses (ranging from “never” to “always”). A total of 10 to 50 points will be given [19]. People with a score below 20 are likely to be mentally healthy, those with a score between 20 and 24 are likely to have a mild mental disorder, those with a score between 25 and 29 are likely to have a moderate mental disorder, and those with a score above 30 are likely to have a severe mental disorder [19]. The third part examined the prevalence of smartphone addiction among the study participants using Smartphone Addiction Scale. The Smartphone Addiction Scale is a 10-item scale that has been validated and has excellent reliability and validity for assessing smartphone addiction. It is a 6-point Likert scale with responses ranging from 1: ‘’strongly disagree’’ to 6: ‘’strongly agree’’. Sixty is the maximum attainable score [11]. The Arabic version for Smartphone Addiction Scale was previously validated for its use to assess smartphone addiction [6].

### Face validity evaluation and piloting phase

The Arabic questionnaires’ clarity and readability were evaluated by expert clinicians. They affirmed that the questionnaire items are straightforward and in line with the study’s aims. Following this, a brief pilot study was conducted on a group of university students. The pilot study affirmed that the questionnaire items are straightforward and simple to complete. Scale reliability analysis for the Arabic version of Kessler Psychological Distress Scale (K10) showed satisfactory results that demonstrate good internal consistency and reliability (Cronbach’s α = 0.88) [20]. Besides, the Arabic version of Smartphone Addiction Scale exhibited adequate reliability and convergent and concurrent validity [6].

### Statistical analysis

Continuous data were represented as mean and standard deviation (sd) because they were normally distributed, as determined by the histogram and skewness and kurtosis measures. We presented categorical variables using frequency and percentage. Using binary logistic regression, predictors of severe psychological distress and smartphone addiction were identified. 30 was the threshold for identifying the dummy variable in the binary logistic regression analysis to identify predictors of psychological distress, and smartphone addiction score of 38.7 was used to to identify predictors of smartphone addiction. The independent variables in the binary logistic regression analysis model were participants’ demographic characteristics and the cut-off points (30 for psychological distress and 38.7 for smartphone addiction) mentioned above were used to identify the dependent variables. A 95% confidence interval (p < 0.05) and a 5% significance level were employed to indicate the statistical significance of the results.

## Results

### Participants’ demographic characteristics

A total of 2337 university students participated in this study. The vast majority of them (87.2%) were females and single (93.8%). More than half of them (71.1%) were aged 21–23 years. Almost half of them (48.1%) were in the first year of their study. Around 62.1% of them were studying non-medical fields. Almost half of them (49.5%) reported that their monthly income category is less than 500 JD. More than half of them (62.9%) reported that “their mental abilities have been negatively affected by the use of smart phones”, and 67.0% reported that “using smartphones has affected your sleep and made it harder to fall asleep”. More than half of them (57.1%) reported that “they feel that everything requires effort and fatigue, and they do not want to do any activity that requires effort”. Table 1 presents the demographic characteristics of the study participants.

**Table 1:** Participants’ demographic characteristics

Variable	Frequency	Percentage
**Gender**		
Females	2037	87.2%
**Age group**		
18–20 years	1661	71.1%
21–23 years	499	21.4%
24–26 years	96	4.1%
27–29 years	42	1.8%
30 years and over	39	1.7%
**Marital status**		
Single	2192	93.8%
Married	119	5.1%
Divorced	20	0.9%
Widowed	6	0.3%
**Study level**		
First year	1123	48.1%
Second year	572	24.5%
Third year	311	13.3%
Fourth year	179	7.7%
Fifth year	59	2.5%
Sixth year (Dentistry, Pharmacy, and Medicine)	21	0.9%
Higher education	72	3.1%
**Faculty**		
Medical sciences	886	37.9%
Non-medical sciences	1451	62.1%
**Monthly income**		
Less than 500 JD	1156	49.5%
500–1000 JD	791	33.8%
1000–1500 JD	208	8.9%
1500 JD and above	182	7.8%
**Do you feel that your mental abilities have been negatively affected by the use of smart phones?** (Yes)	1106	62.9%
**Do you feel that using smartphones has affected your sleep and made it harder to fall asleep?** (Yes)	1177	67.0%
**I feel that everything requires effort and fatigue, and I do not want to do any activity that requires effort**. (Yes)	1335	57.1%

### Psychological distress profile among the study participants

**Fig. 1:**
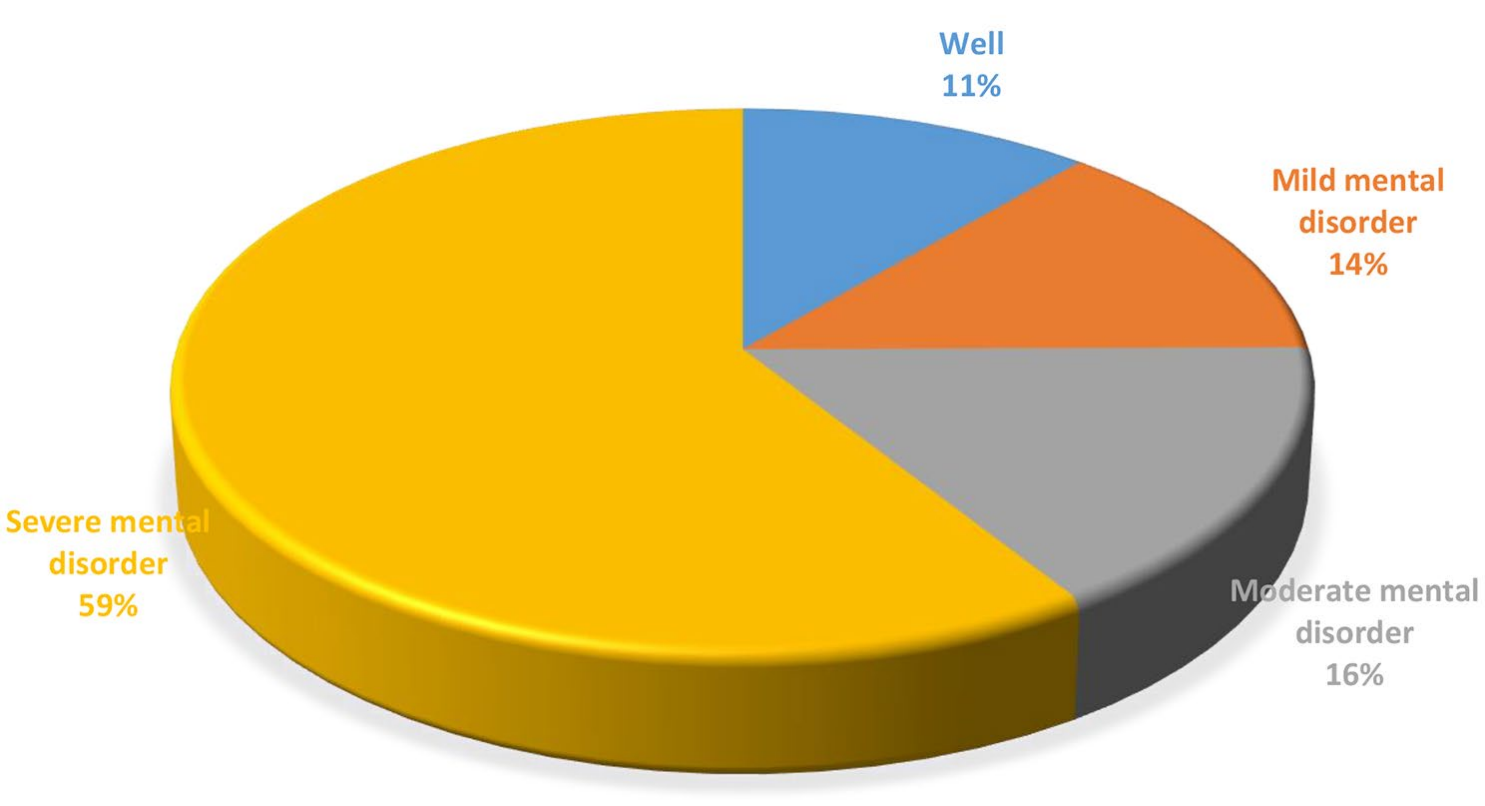
Psychological distress profile of the study participants

The mean psychological distress score for the study participants was 30.0 (SD: 8.9). The majority of the study participants (88.7%) showed mental disorder state to different degree of severity according to their score on Kessler psychological distress scale score. More than half of the study participants (59.1%) had a psychological distress score of 30 and above, which indicates a severe mental disorder state. Figure 1 presents the psychological distress profile of the study participants.

The study participants’ responses to questions about anxiety and depressive symptoms that they have experienced in the most recent 4-week period are presented in Table 2. Participants reported that they have experiences anxiety and depressive symptoms all the time to different proportion that ranged between 6.8 and 25.4%. The most commonly reported anxiety and depressive symptom was that they feel that everything was an effort (25.4%). The least commonly reported anxiety and depressive symptom was that they you feel so restless you could not sit still (6.8%).

**Table 2:** Participants’ response to Kessler psychological distress scale

Number	Item	None of the time	A little of the time	Some of the time	Most of the time	All of the time
1	During the last 30 days, about how often did you feel tired out for no good reason?	6.2%	15.6%	25.0%	34.4%	18.7%
2	During the last 30 days, about how often did you feel nervous?	6.1%	16.6%	28.4%	33.3%	15.6%
3	During the last 30 days, about how often did you feel so nervous that nothing could calm you down?	28.8%	27.2%	22.7%	14.2%	7.0%
4	During the last 30 days, about how often did you feel hopeless?	12.2%	21.2%	25.0%	22.3%	19.3%
5	During the last 30 days, about how often did you feel restless or fidgety?	8.0%	22.9%	29.9%	26.0%	13.2%
6	During the last 30 days, about how often did you feel so restless you could not sit still?	31.6%	26.5%	22.8%	12.2%	6.8%
7	During the last 30 days, about how often did you feel depressed?	12.8%	19.3%	23.0%	22.7%	22.2%
8	During the last 30 days, about how often did you feel that everything was an effort?	6.4%	17.1%	23.9%	27.2%	25.4%
9	During the last 30 days, about how often did you feel so sad that nothing could cheer you up?	16.3%	22.1%	23.2%	20.3%	18.1%
10	During the last 30 days, about how often did you feel worthless?	39.0%	21.3%	15.9%	12.1%	11.8%

### Smartphone addiction scale

The mean smartphone addiction score for the study participants was 38.7 (SD: 11.3) out of 60 (which is equal to 64.5%). More than half of the study participants (56.7%) had a smartphone addiction score of 30 and above, which reflects a smartphone addiction state. The study participants’ responses to statements that examined their dependence and addiction to smartphone are presented in Table 3. Participants reported their agreement (answered either agree or strongly agree) to different statements that examined their dependence and addiction to smartphone to different proportion that ranged between 30.4 and 64.3%. The most commonly agreed upon statement was that they are using their smartphone longer than they had intended (64.3%). The least commonly agreed upon statement was that they will never give up using their smartphone even when their daily life is already greatly affected by it.

**Table 3:** Participants’ responses to smartphone addiction scale

Number	Item	Strongly disagree	Disagree	Somewhat disagree	Somewhat agree	Agree	Strongly agree
1	Missing planned work due to smartphone use	8.0%	10.9%	8.1%	20.0%	30.6%	22.6%
2	Having a hard time concentrating in class, while doing assignments, or while working due to smartphone use	6.5%	14.0%	8.2%	18.6%	32.1%	20.5%
3	Feeling pain in the wrists or at the back of the neck while using a smartphone	9.1%	16.6%	8.0%	20.1%	31.5%	14.7%
4	Won’t be able to stand not having a smartphone	8.6%	16.3%	7.8%	16.5%	28.2%	22.5%
5	Feeling impatient and fretful when I am not holding my smartphone	12.4%	20.4%	9.2%	15.7%	25.8%	16.5%
6	Having my smartphone in my mind even when I am not using it	13.8%	23.3%	10.0%	15.9%	23.1%	13.8%
7	I will never give up using my smartphone even when my daily life is already greatly affected by it.	18.5%	24.8%	10.8%	15.4%	19.7%	10.7%
8	Constantly checking my smartphone so as not to miss conversations between other people on Twitter or Facebook	14.7%	21.4%	9.2%	16.7%	24.8%	13.2%
9	Using my smartphone longer than I had intended	5.4%	10.1%	6.5%	13.8%	36.6%	27.7%
10	The people around me tell me that I use my smartphone too much.	11.5%	21.2%	9.0%	14.5%	24.0%	19.8%

### Predictors of psychological distress and smartphone addiction

Females, divorced, those who feel that their mental abilities have been negatively affected by the use of smart phones, those who feel that using smartphones has affected their sleep and made it harder to fall asleep, and those feel that everything requires effort and fatigue, and they do not want to do any activity that requires effort were more likely to have severe psychological distress compared to others (p < 0.05). On the other hand, participants with income category 500–1000 JD were 27.0% less likely to have severe psychological distress compared to others (p < 0.001).

Females, those who feel that using smartphones has affected their sleep and made it harder to fall asleep, and those feel that everything requires effort and fatigue, and they do not want to do any activity that requires effort were more likely to be smartphone addicted compared to others (p < 0.05). On the other hand, participants who are aged 30 years and over, those who are married, completing their higher education, and have an income category of 1500 JD and above were less likely to be smartphone addicted compared to others (p < 0.05), Table 4.

**Table 4:** Binary logistic regression analysis

Variable	Odds ratio of having severe psychological distress (95% confidence interval)	Odds ratio of being smartphone addicted (95% confidence interval)
**Gender**		
Females (reference category)	1.00	
Males	0.52 (0.41–0.66)***	0.63 (0.49–0.80)***
**Age group**		
18–20 years (reference category)	1.00	
21–23 years	0.94 (0.77–1.15)	0.94 (0.77–1.15)
24–26 years	0.97 (0.64–1.47)	1.02 (0.68–1.55)
27–29 years	0.84 (0.45–1.54)	0.96 (0.55–1.88)
30 years and over	0.73 (0.39–1.37)	0.47 (0.25–0.90)*
**Marital status**		
Single (reference category)	1.00	
Married	1.06 (0.73–1.55)	0.69 (0.48-1.00)*
Divorced	3.96 (1.16–13.55)*	1.15 (0.47–2.81)
Widowed	0.14 (0.02–1.18)	1.53 (0.28–8.35)
**Study level**		
First year (reference category)	1.00	
s year	0.97 (0.80–1.18)	1.16 (0.96–1.40)
Third year	0.93 (0.73–1.18)	1.17 (0.92–1.50)
Fourth year	1.14 (0.83–1.56)	1.15 (0.84–1.56)
Fifth year	0.94 (0.56–1.59)	1.12 (0.66–1.89)
Sixth year (Dentistry, Pharmacy, and Medicine)	1.39 (0.56–3.45)	0.69 (0.29–1.63)
Higher education	1.40 (0.85–2.30)	0.57 (0.35–0.91)*
**Faculty**		
Medical sciences (reference category)	1.00	
Non-medical sciences	1.16 (0.98–1.37)	1.04 (0.88–1.24)
**Monthly income**		
Less than 500 JD (reference category)	1.00	
500–1000 JD	0.73 (0.62–0.87)***	1.12 (0.94–1.33)
1000–1500 JD	0.77 (0.58–1.03)	1.04 (0.78–1.39)
1500 JD and above	1.01 (0.74–1.38)	0.73 (0.54–0.99)*
**Do you feel that your mental abilities have been negatively affected by the use of smart phones?** (Yes)	1.92 (1.58–2.34)***	5.73 (4.53–7.25)***
**Do you feel that using smartphones has affected your sleep and made it harder to fall asleep?** (Yes)	1.84 (1.50–2.25)***	3.46 (2.76–4.33)***
**I feel that everything requires effort and fatigue, and I do not want to do any activity that requires effort**. (Yes)	3.25 (2.56–4.12)***	6.91 (5.39–8.86)***

## Discussion

Excessive problematic usage of smart phones and addiction to the usage of these devices need to be substantially understood, where it affects the physical and mental health negatively [4, 13, 17]. This study aimed to explore smart phone addiction and its mental health risks among university students in Jordan by understanding their psychological distress profile, study their smartphone addiction scale, and detect the predictors of psychological distress and smartphone addiction.

Mental disorders are a serious public health issue in Jordan [21, 22], in this study, 88.7% of the study participants showed mental disorder state with different degree of severity according to their score on Kessler psychological distress scale score, where more than half of the study participants (59.1%) had a psychological distress score of 30 and above, which indicates a severe mental disorder state, this is in line with previous studies, where the prevalence of severe mental and psychological disorders are significantly high among adolescents and university students in Jordan [21, 23], and the reason for this increased prevalence of mental disorders is the presence of a stigma of seeking help in mental distress in Arab countries [24], and instead of seeking treatment, university students prefer dealing with stress alone, believe that it is normal having stress in university, and do not see that their need as serious [25].

In this study, participants reported that they have experiences anxiety and depressive symptoms all the time with different proportions that ranged between 6.8 and 25.4%, where the most commonly reported anxiety and depressive symptom was that they feel that everything was an effort (25.4%). Indeed, 75.0% Jordanian women reported this symptom of anxiety and depression as the most reported complaints [26]. Among the study participants 56.7% of the participants had a smartphone addiction score of 30 and above, which reflects a smartphone addiction state, this high prevalence of addiction toward using smart phones is highly related to multiple factors including that smart phone is a tool for passing time as well as it became an essential daily needed application, and this is what led to increase the presence of smart phone addiction concept [27], substantially, the increase prevalence of different social media platforms and services is a main reason for increased smart phone addiction [28], while the usage of social media networks believed to be addictive, the overuse of social media should be treated as public health concern while dealing with smart phone addiction [27, 28].

As an indicator for excessive problematic usage of smart phone and smart phone addiction within the study participants, 64.3% of them agreed upon the statement that they are using their smartphone longer than they had intended, this is in line with the study among university students that shows 71.9% agreed on the same statement that they are using their smart phone longer than they intended to [29], indeed, it is also consistent to our findings that the least commonly agreed upon statement was that they will never give up using their smartphone even when their daily life is already greatly affected by it. Multiple factors including gender, age, marital status, mental status, and income were found to have a significant impact as a predicator of psychological distress and smartphone addiction. This study found that females, divorced, those who feel that their mental abilities have been negatively affected by the use of smart phones, those who feel that using smartphones has affected their sleep and made it harder to fall asleep, and those feel that everything requires effort and fatigue, and they do not want to do any activity that requires effort were more likely to have severe psychological distress compared to others. Indeed, female students have higher susceptibility to report mental distress than male university students [30], where this is related to the natural response of females toward any psychological modulator [31]. Meanwhile, being divorced women is directly associated to psychological distress that results from excessive use of smart phone and smart phone addiction, where it is believed dealing with stress alone is a major cause for the increased psychological distress among university students [25], as well as, smart phone addiction affect the sleep status, where it make it harder to fall asleep and affect the quality of the sleep [27, 28], also smart phone usage addiction negatively affect the mental health of youth as it interfere with their daily life pattern [32], where these factors consequently effect the mental status and increase the psychological distress. On the other hand, participants with income category 500–1000 JD were 27.0% less likely to have severe psychological distress compared to others, where intermediate to high income population are less likely to develop mental distress than lower income population, where the role of stressor highly affect lower income population and it is directly associated with psychological distress [33].

In fact, the same factors that predicate the psychological distress found to effect smart phone addiction, where females, those who feel that using smartphones has affected their sleep and made it harder to fall asleep, and those feel that everything requires effort and fatigue, and they do not want to do any activity that requires effort were more likely to be smartphone addicted compared to others, indeed, regarding smart phone addiction gender differences, women were more dependence upon using their smart phones than men, and their use is for multiple purposes including social relationship usage and interpersonal motives [34]. Still, excessive usage of smart phone is highly related to sleep disorders including difficulty falling asleep [27], also, related to mental distress by making it harder to feel doing things that they have to do and interfere with their daily activity [29, 32], and these factors predicate smart phone addiction along with psychological distress. On the other hand, participants who are aged 30 years and over, those who are married, completing their higher education, and have an income category of 1500 JD and above were less likely to be smartphone addicted compared to others, regarding age 30 and over, there is a relation between the increased age of students and decreased usage of their smart phones where students over 20 years old are less likely to develop smart phone addiction than younger students [35], as well as, being in a marriage relationship reduces the need for excessive smart phone usage since the relationship is established in close distance basis and no need for male or female to maintain or looking for a relationship through their smart phones [36], and also having a partner to rely on decreases the mental distress risks [25], meanwhile, high income in our study finding was less likely to be smart phone addiction, while these finding contradict other study findings, where it shows that students from a high income families and backgrounds were more likely to use their phones higher than those from low income families [37], however, other studies shows an equal opportunity to smart phone usage regardless of income status [38, 39].

In the matter of fact, using smart phones became essential nowadays and students depend on the usage of their smart phones on a daily basis, there is no need to stop using smart phones at all, but there is an urgent need to stop the excessive problematic usage of smart phones that substantially lead to smart phone addiction and alter the psychological status among university students, more control on the usage of smart phones among university students need to be implemented, awareness campaign may also be useful to spread the awareness and mitigate smart phone addiction risks and it physical and mental health consequences.

## Conclusion

Excessive problematic usage of smartphones and addiction to these devices have a significant negative impact on both physical and mental health. This study aimed to explore smartphone addiction and its mental health risks among university students in Jordan. The findings revealed that mental disorders are a serious public health issue in Jordan, with a high prevalence among university students. The stigma surrounding seeking help for mental distress contributes to the increased prevalence of mental disorders in this population. Several factors were found to be predictors of both psychological distress and smartphone addiction, including gender, age, marital status, mental status, and income. Females, those who felt that their mental abilities were negatively affected by smartphone use, and those who experienced difficulty falling asleep and fatigue were more likely to have severe psychological distress and smartphone addiction. While smartphones have become essential in daily life, it is crucial to address the excessive problematic usage that leads to addiction and negatively impacts the psychological well-being of university students. Implementing control measures and raising awareness about the risks of smartphone addiction can help mitigate its physical and mental health consequences.

## Data Availability

The datasets supporting the conclusions of this study are available from the corresponding author upon request.

## Abbreviations

K10: Kessler Psychological Distress Scale
SD: standard deviation
SPSS: Statistical package for social sciences
CI: Confidence interval
